# Increased *THEMIS* First Exon Usage in CD4+ T-Cells Is Associated with a Genotype that Is Protective against Multiple Sclerosis

**DOI:** 10.1371/journal.pone.0158327

**Published:** 2016-07-20

**Authors:** Jessica L. Davies, Sara Thompson, Harpreet Kaur-Sandhu, Stephen Sawcer, Alasdair Coles, Maria Ban, Joanne Jones

**Affiliations:** Department of Clinical Neurosciences, University of Cambridge, Cambridge, United Kingdom; University of Lisbon, PORTUGAL

## Abstract

Multiple sclerosis is an autoimmune disease of the central nervous system. Genome wide association studies have identified over 100 common variants associated with multiple sclerosis, the majority of which implicate immunologically relevant genes, particularly those involved in T-cell development. SNP rs13204742 at the *THEMIS/PTPRK* locus is one such variant. Here, we have demonstrated mutually exclusive use of exon 1 and 2 amongst 16 novel *THEMIS* isoforms. We also show inverse correlation between *THEMIS* expression in human CD4+ T-cells and dosage of the multiple sclerosis risk allele at rs13204742, driven by reduced expression of exon 1- containing isoforms. *In silico* analysis suggests that this may be due to cell-specific, allele-dependent binding of the transcription factors FoxP3 and/or E47. Research exploring the functional implications of GWAS variants is important for gaining an understanding of disease pathogenesis, with the ultimate aim of identifying new therapeutic targets.

## Introduction

Multiple sclerosis (MS) is the most common cause of chronic neurological disability in young adults. Although disease aetiology is uncertain, it is clear that both genetic and environmental factors influence an individual’s risk of disease development[1].

Genome wide association studies (GWAS)[2] and subsequent fine mapping of risk loci (Immunochip)[3] have identified 110 common MS risk variants, the majority of which lie within 50kb of genes with immunological function, particularly genes involved in T-cell development[2].

One of the implicated genomic regions lies on chromosome 6q and contains two genes critically important for T-cell development: *thymocyte expressed molecule involved in selection* (*THEMIS*) and *protein tyrosine phosphatase*, *receptor type*, *κ* (*PTPRK*). Association with this locus was first identified in the 2011 GWAS, in which the most associated SNP was rs802734, an intergenic SNP located 56 kb 5’ of *THEMIS* and 11 kb 3’ of *PTPRK* [2]. Subsequent fine-mapping of this region in the Immunochip study identified rs13204742 as the lead SNP, a variant 23 kb 5’of *THEMIS*, and 44 kb 3’ of *PTPRK* [3]. Notably, an association between SNP rs802734 and coeliac disease has also been demonstrated[4], and subsequent investigations have shown that this SNP influences *THEMIS*, but not *PTPRK*, expression in treated coeliac disease patients[5]. Fine-mapping data for coeliac disease localised the causal variants driving this association close to the 3’ UTR of *PTPRK* [6].

SNP rs13204742 is not in LD with any common coding variants in either *THEMIS* or *PTPRK*, implying that this variant likely influences disease risk by altering expression rather than by altering protein structure and/or function[7]. This region also contains a non-coding antisense RNA (*RP11-103C16*.*2*) which overlaps *PTPRK* (Ensembl.org). Between 50–70% of loci in mammalian genomes are said to code for antisense transcripts, which are proposed to regulate expression of their sense genes[8].

Both THEMIS and PTPRK are required for thymopoieisis, which has been shown to be deficient in MS[9–11]. In particular they are required for thymocyte positive selection and commitment of double positive (DP) T-cells to the CD4+ lineage, including to the T regulatory cell (Treg) lineage. Tregs from individuals with MS are widely reported to have impaired suppressive capacity[12], however it has been suggested that this may be due to inclusion of activated CD4+CD25hiCD127hi effector T-cells in the original Treg assays [13]. Reduced numbers of recently thymically derived Tregs have been reported by some groups [14, 15]. Rodents deficient in either *THEMIS* or *PTPRK* have fewer CD4+ T-cells[16–21] and lack CD4+ Treg function[22]. Murine and human studies have also shown that THEMIS and PTPRK play a key role in the TCR signalling pathway[16, 23, 24]. Unlike THEMIS[19], PTPRK is also expressed at high level in B-cells, in which it acts as a tumour suppressor[25].

Therefore, given the reported immunological functions of THEMIS and PTPRK, we elected to investigate the effects of SNP rs13204742 on T-cell gene expression at the *THEMIS/PTPRK* locus, thymic function, the peripheral T-cell repertoire, and T-cell activation.

## Results

### *Ex vivo* CD4+ and CD8+ T-cell *THEMIS* mRNA expression decreases with increasing genetic risk

It is known that genomic exon 5 of *THEMIS* encodes one of its two CABIT domains, a proline rich region (PRR) required for protein-protein interactions, and a YY-motif required for interaction with Grb2, making it crucial to the function of THEMIS[26]. We therefore first explored genotypic effects on the mRNA expression of (i) “functional” exon 5-containing *THEMIS* isoforms, (ii) *PTPRK* and (iii) the antisense non-coding RNA gene *RP11-103C16*.*2* in *ex vivo* CD4+ and CD8+ T-cells and CD19+ B-cells.

**Fig 1:**
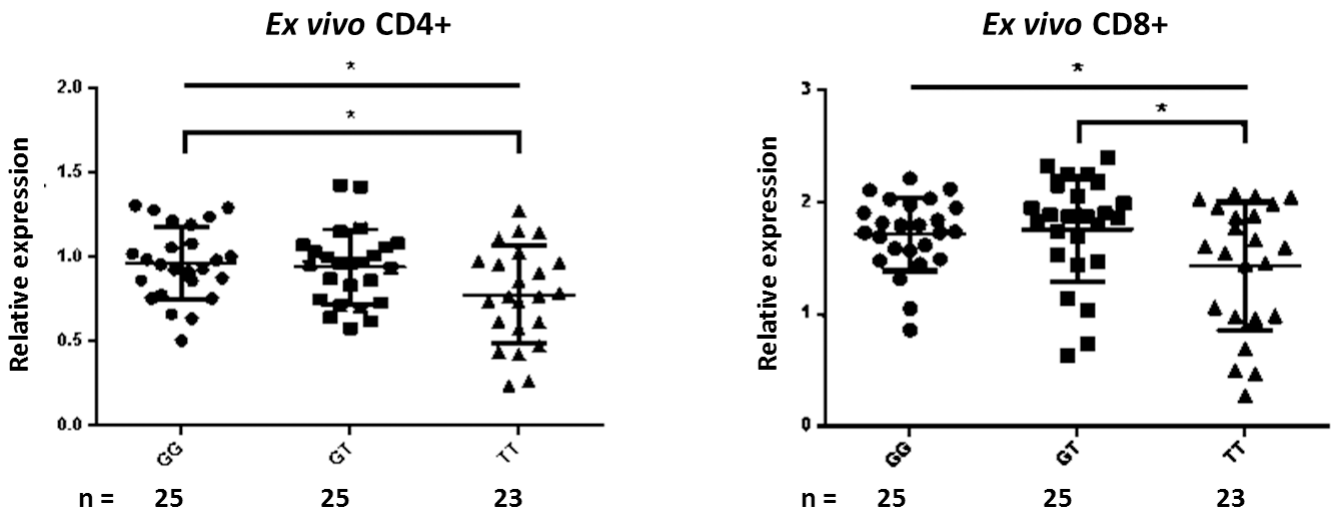
*THEMIS* expression is decreased in *ex vivo* CD4+ and CD8+ T-cells from risk homozygotes. Error bars display standard deviation from the mean. Upper horizontal lines indicate the significance level of ANOVA tests; lower horizontal brackets represent significant differences identified by Turkey’s multiple comparisons tests between the bracketed genotypes. * p ≤ 0.05.

Decreased exon 5 *THEMIS* mRNA expression in *ex vivo* CD4+ and CD8+ T-cells with increased risk allele dosage was observed (n = 73) (CD4+ ANOVA p = 0.018, Turkey’s test ‘GG vs TT’ p = 0.024, ‘GG vs GT’ p = 0.943, ‘GT vs TT’ p = 0.053; CD8+ ANOVA p = 0.036, Turkey’s test ‘GG vs TT’ p = 0.094, ‘GG vs GT’ p = 0.940, ‘GT vs TT’ p = 0.044) (Fig 1). *THEMIS* was detected in B-cells, but no genotypic differences were seen (data not shown), suggesting a T-cell-specific effect. We noted that *THEMIS* expression was higher in CD8+ than CD4+ T-cells, as has been previously reported in mice [19]. *RP11-103C16*.*2* mRNA expression was inversely related to *PTPRK* expression (data not shown), in keeping with the hypothesis that it regulates sense gene transcription. No genotypic differences in *PTPRK* or *RP11-103C16*.*2* expression were observed in *ex vivo* CD4+ and CD8+ T-cells or B-cells (Figure A in S1 File); genotype was also not correlated with the ratio of *RP11-103C16*.*2/PTPRK* expression in *ex vivo* CD4+ and CD8+ T-cells (Figure A in S1 File).

### Identification of novel *THEMIS* isoforms

**Fig 2:**
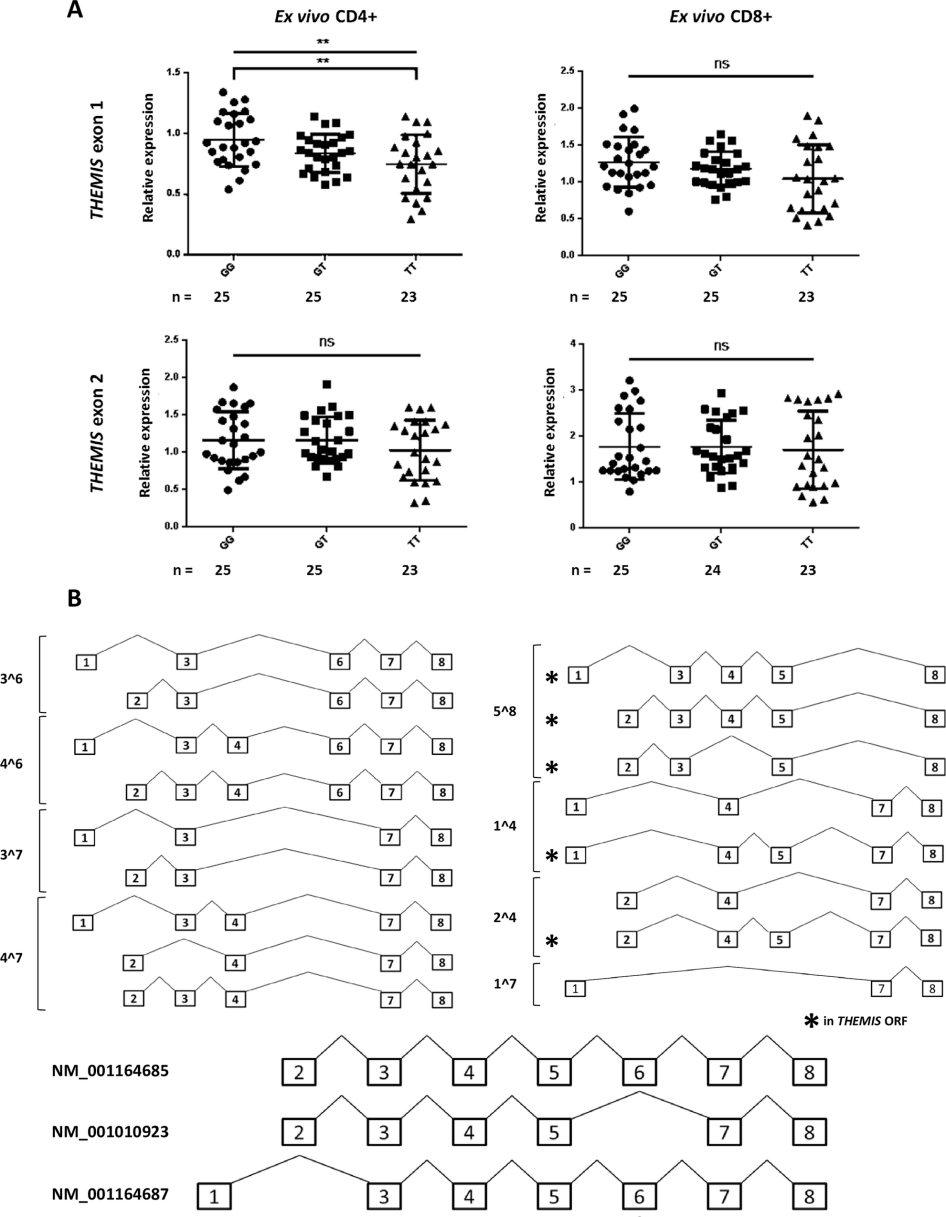
Genotype at SNP rs13204742 is associated with differences in T-cell *THEMIS* exon 1 expression. (A) Decreased *THEMIS* exon 1 expression in *ex vivo* CD4+ and CD8+ T-cells is associated with an increasing genetic load of the risk allele (T). There are no genotypic differences in *THEMIS* exon 2 expression. ** p ≤ 0.01; ns = non-significant. (B) Exon structures of 16 novel *THEMIS* isoforms, grouped by novel exon boundaries; RefSeq isoforms are also displayed with their RefSeq identifiers (bottom).

Three isoforms of *THEMIS* are listed in the RefSeq database (www.ncbi.nlm.nih.gov/refseq/, accessed on March 31^st^ 2015); two use genomic exon 2 as their first exon (NM_001010923 and NM_001164685), the other uses genomic exon 1 (NM_001164687) (Fig 2). This raises the possibility that *THEMIS* expression may be regulated in time and/or location by alternative first exon usage, a mechanism proposed to be a consequence of differential transcription factor recruitment[27–29].

To explore this further, we first searched for novel *THEMIS* exon junctions by PCR and then validated these by Sanger sequencing of PCR products. Using combinations of exon-spanning primers, intra-exonic primers, and internal sequencing primers, a total of 16 novel isoforms were inferred from sequencing of the PCR products (Fig 2B) (primer sequences given in Table A in S2 File). *In silico* analysis (NCBI ORF finder) suggested that only 5 of these isoforms were in open reading frame (ORF) for THEMIS (Table B in S2 File). Alternative first exon usage was demonstrated in all isoforms identified–exon 1 and 2 were always mutually exclusive.

Of the 16 isoforms, 11 lacked exon 5, which is proposed to code for key functional domains of THEMIS[26]. The original primers we used to investigate “functional” *THEMIS* expression by qPCR spanned the exon 4/5 junction, suggesting that they were limited in their interrogation of total *THEMIS* expression.

### Decreased *THEMIS* expression is due to a decrease in exon 1-containing isoforms

Hypothesising that rs13204742 might exert its regulatory effects by altering the balance of exon 1 and exon 2-containing *THEMIS* isoforms, we tested for genotypic associations with differences in first exon usage.

A significant decrease in *THEMIS* exon 1 expression was observed in *ex vivo* CD4+ T-cells, with increasing genetic risk (ANOVA p = 0.0055; Turkey’s test, ‘GG vs TT’ p = 0.0038). A similar trend was observed in CD8+ T-cells, but did not reach significance (ANOVA p = 0.1160; Turkey’s test ‘GG vs TT’ p = 0.0958) (Fig 2A). There was no association between *THEMIS* exon 2 expression and genotype (CD4+ ANOVA p = 0.3588; CD8+ Kruskal-Wallis p = 0.7427). These data suggest that the genotype-associated decrease in *THEMIS* expression identified using exon 4/5 spanning primers was due to reduced expression of *THEMIS* exon 1-containing isoforms. (Sequencing electropherograms are displayed in Figure B of S1 File). This observation is supported by the significant positive correlation between *THEMIS* exon 1 and exon 4/5 expression (Figure C of S1 File).

### A trend towards differences in *THEMIS* exon 1 expression were observed at the protein level

*Ex vivo* CD4+ T-cells exhibiting the most extreme levels of both *THEMIS* exon 1 and exon 2 expression by qPCR were selected for analysis of THEMIS protein expression (n = 6, 3 vs. 3). Those individuals with greatest *THEMIS* mRNA expression showed a trend towards increased THEMIS protein expression, as determined by western blotting (Figure D of S1 File). These data suggest that genotypic differences in *THEMIS* mRNA expression may be maintained at the protein level.

### Functional consequences of genetic variation at SNP rs1320472 are not associated with differences in T-cell *IL2* expression, or thymic activity

Given their role in TCR signalling, we next explored the influence of genotype on “functional” *THEMIS*, *THEMIS* exons 1 and 2, *PTPRK*, and *IL2* expression resulting from CD3/CD28 induced T-cell activation in separated CD4+ and CD8+ T-cells. Fold differences in *THEMIS*, *PTPRK* and *IL2* gene expression upon stimulation were determined at the peak/trough of expression post-stimulation, and the time-point of return to baseline expression was also determined. TCR stimulation did not reveal genotypic differences in *THEMIS*, *PTPRK* or *IL2* mRNA expression (data not shown).

The association between genotype at SNP rs13204742 and thymic function was investigated by quantification of sjTRECs/ml and sjTRECs/μg CD4+ and CD8+ DNA, and by examining the relative frequency of circulating naïve CD4+ and CD8+ T-cells (CCR7+CD45RA+) and recent thymic emigrants (RTEs; defined as CD4+CCR7+CD45RA+CD31+ T-cells). No genotypic difference was found (n = 73; Figure E of S1 File). Furthermore, no differences were found in the proportion of circulating naïve Tregs (CD4+CD45RA+FoxP3+), memory Tregs (CD4+CD45RA-FoxP3hi) or RTE Tregs (CD4+CD45RA+FoxP3+CD31+) by genotype (n = 32; Figure F of S1 File).

### *In silico* analysis of transcription factor binding and chromatin state

The mechanism by which SNP rs13204742 regulates *THEMIS* mRNA expression was explored by *in silico* analyses of transcription factor binding and chromatin state at this SNP and proximal SNPs rs67707912 and rs72973730 (r^2^ ≥ 0.8 with rs13204742).

According to Alggen PROMO, the minor risk alleles were predicted to disrupt binding of transcription factors E47 and FoxP3 at SNP rs13204742 and SNP rs67707912 respectively; transcription factor binding at SNP rs72973730 was not disrupted.

Haploreg v4.1 data (www.broadinstitute.org/mammals/haploreg/), from the Roadmap Epigenomics Consortium, 2015, were used to investigate chromatin state at SNPs rs13204742, rs67707912 and rs72973730. Promoter and enhancer marks (H3K4me3 and H3K4me1, respectively) were present at all three SNPs in primary T-cell subsets. The H3K27ac mark (distinguishing active from poised/inactive enhancers[30]) was detected at SNPs rs67707912 and rs72973730 across T-cell subsets, but limited to PMA and ionomycin stimulated T-cells and naïve CD8+ T-cells at SNP rs13204742. From these data, an active regulatory function could be conferred to all three SNPs.

## Discussion

To date, 110 common variants have been shown to be associated with susceptibility to multiple sclerosis[3], and much current research is aimed at understanding the functional effects of these variants. Here, we explored the functional mechanisms underlying SNP rs13204742, the most significantly associated SNP at the *THEMIS/PTPRK* locus in the Immunochip study[3]. We focused our investigations on Immunochip SNP rs13204742, yet dense genotyping in celiac disease and multiple sclerosis has revealed multiple independent signals at the *THEMIS/PTPRK* locus[3, 6] so further work will be required to refine the association signals within this region.

We have demonstrated that e*x vivo* CD4+ T-cell *THEMIS* expression shows a significant inverse correlation with carriage of the multiple sclerosis risk allele, and that this correlation is also reflected in protein expression; we found an equivalent trend in CD8+ T-cells. We have shown that this is due to decreased expression of exon 1-containing *THEMIS* isoforms. *In silico* analysis suggested that this may result from allele dependent binding of the transcription factors FoxP3 and/or E47.

We did not find any evidence that decreased expression of *THEMIS* exon 1-containing isoforms influences thymopoiesis (either of conventional T-cells or Tregs) as has been previously reported in murine studies[16, 17, 19, 20]. However, we have been limited to indirect measurements of thymic function–namely quantification of TRECs and the number of circulating recent thymic emigrants, which may be insufficiently sensitive to detect small genotype driven differences in thymic function.

Subtle defects in proximal TCR signalling, specifically impaired Erk and calcium signalling, have been observed in anti-CD3 and anti-CD4 activated thymocytes from Themis deficient mice[16]. However, in our analysis we found no evidence that genotype influences TCR-induced *IL2* expression, although our experiments likely had a low detection sensitivity; differences may have been observed under different stimulation conditions. It is also possible that genotypic effects on both thymic function and TCR signalling may be masked by subject differences in environmental exposures (such as infections) and genetic risk at other established MS-associated loci.

We have identified 16 new isoforms of *THEMIS*, of which a significant proportion (n = 11) were not in open reading frame (ORF); the reason why so many untranslated mRNAs should be produced is not clear, but may represent an additional level of control of protein expression[31, 32]. The five isoforms in ORF used either exon 1 or exon 2 as the first exon, in a mutually exclusive manner. Alternate first exon usage has been shown to be a mechanism of regulating gene expression in time and/or location as a consequence of differential transcription factor recruitment, and we found evidence that genotype at rs13204742 influences the expression of exon 1-containing isoforms[27–29]. Further experimental work would be required to confirm the role of alternate first exon usage in regulating *THEMIS* expression.

*In silico* analysis suggested that genotype at SNP rs13204742 and SNP rs67707912 affect binding of the transcription factors E47 and FoxP3, respectively. SNP rs67707912 was selected for analysis as it is in strong LD with SNP rs13204742 and, despite SNP rs13204742 having been initially identified in the Crohn’s Immunochip[33], rs67707912 was shown to be the likely associated SNP at the *THEMIS/PTPRK* locus by subsequent algorithm-based analysis[34]. The association of SNP rs13204742 and its proximal SNPs with active promoter and enhancer marks in T-cell subsets provides additional evidence of their regulatory function. Chromatin immunoprecipitation (ChIP) and chromatin conformation capture (3C) studies would be needed to establish transcription factor binding and confirm chromatin interactions of these SNPs with their target gene(s).

In summary, this is the first study to investigate the effects of genotype at SNP rs13204742 on immune cell function. Our data suggest that the multiple sclerosis risk allele is associated with decreased *THEMIS* expression in human T-cells, and that this association is driven by differences in exon 1-containing *THEMIS* isoforms. Research exploring the functional implications of GWAS variants, such as those performed here, is important for gaining an understanding of the pathways involved in disease with the ultimate aim of identifying new therapeutic targets.

## Materials and Methods

### Participant recruitment

Healthy volunteers (n = 73) were recruited via the Cambridge BioResource and consented to give blood for research purposes under CAMSAFE. CAMSAFE was given ethical approval by the NRES Committee East of England–Cambridge Central (REC 11/33/0007). Written consent was obtained for study participation, as approved by the ethics committee. Individuals were matched for age and gender across genotypes (see Table 1). Samples were processed blinded to genotype. Individuals taking immunotherapies, or with an active infection at the time of donation, were excluded. Healthy individuals, as opposed to multiple sclerosis patients, were studied to remove the complexities and confounding influences of an autoreactive immune system on the function of SNP rs13204742.

**Table 1:** Participant demographics.

Genotype	GG	GT	TT
**Average age**	42	43	42
**Gender (F:M)**	20:5	19:6	19:4
**N**	25	25	23

### Cell isolation and separation

PBMCs were isolated from heparinised blood by Ficoll density gradient centrifugation (Ficoll PaquePlus; GE Healthcare, Amersham), and magnetically separated by positive selection to isolate CD19+ B-cells, followed by Pan T-cell isolation and CD8+ positive selection to enrich for CD4+ T-cells and CD8+ T-cells (Miltenyi Biotec). Purities of enriched cell fractions averaged between 91 and 95% (n = 26; data not shown).

### T-cell activation

1 x 10^6^/ml CD4+ and CD8+ T-cells were separately cultured in RPMI 1640 (Invitrogen) containing 1% penicillin/streptomycin (Sigma) and 10% (v/v) foetal calf serum, at 37°C in 5% CO_2,_ with and without activation by CD3/CD28 Dynabeads (1:1 bead:cell ratio; Life technologies). Cells were harvested after 3, 24, and 72 h.

### Flow cytometric T-cell phenotyping

PBMCs were phenotyped using the following antibodies from BD Biosciences: CD4-V500, CD8-PE, CCR7-FITC, CD45RA-PECy7, CD31-FITC, CD39-APC, CD25-PE, CD127-V450, FoxP3-APC. Intranuclear staining was performed using the FoxP3/Transcription Factor Staining Buffer Set (eBioscience). Data were acquired on a Canto II and analysed using FlowJo v7.6.5 (Tree Star Inc).

### Quantitative real-time PCR

RNA was extracted using Trizol (Invitrogen), and DNase I treated (Fermentas). Average RNA integrity number was 8.9 (Agilent Bioanalyzer; n = 44; data not shown). cDNA was synthesised using the High Capacity cDNA Reverse Transcription Kit with RNase Inhibitor (Applied Biosystems). Semi-quantitative real-time PCR (qPCR) was performed on a QuantStudio™ 6 Flex Real-Time PCR System; cycling conditions were: 95°C 10 min; 40 cycles of 95°C 15 s / 60°C 1 min. Gene expression assays for *THEMIS* (FAM; Hs01041269_m1), *PTPRK* (FAM; Hs00935224_m1), *IL2* (FAM; Hs00174114_m1), and *TBP* (VIC; Hs00427620_m1) were ordered from Applied Biosystems. The following primers and probes were designed using Primer Express v2.0 and ordered from Life technologies: *THEMIS* exon 1 5’-GGCTCTTCCTGGATCCCTTATTT-3’ (forward), 5’-TGCGTAAGAGCACTGGAGCAT-3’ (reverse) and 6FAM-CAGGAGAGGAGGAAAA-MGB (probe); *THEMIS* exon 2 5’-TCACCCAGAAGCCACAAGTTTC-3’ (forward), 5’-CCATTGCTATGCCTTGGGTAGTT-3’ (reverse) and 6FAM-TGAGCACCAGGTCTAC-MGB (probe); *RP11-103C16*.*2* 5’-GGACCACAGCAGGGAAAAAG-3’ (forward), 5’-GCGATGAGCAGTGTGGAGAA-3’ (reverse) and 6FAM-CTGGAGGAAGCTCAC-MGB (probe). Relative expression was achieved using the standard curve method. A standard curve of Ct values for both the reference gene (*TBP*) and target gene was made from stock immune cell cDNA; for each sample loaded in triplicate, the average Ct values of the reference and target gene were determined, and the relative expression level was calculated from the equation of the line for the standard curve. Absence of an effect of cell stimulation on *TBP* mRNA expression was verified. Fold difference in expression upon stimulation was determined relative to an unstimulated cell control.

### End-point PCR and Sanger sequencing

RNA and cDNA were prepared as described above. Primers for *THEMIS* isoform detection are given in Table A of S2 File. Each 10 μl reaction contained 500 nM of each primer, 50 ng cDNA, and AmpliTaq Gold (Applied Biosystems). Cycling conditions used were: 95°C 10 min; 40 cycles of 95°C 30 s / 60°C 30 s / 72°C 30 s; 72°C 7 min. PCR products were sequenced on an Applied Biosystems 3730xl.

### Western blotting

CD4+ T-cells were magnetically isolated from PBMCs by positive selection and cell lysates were stored in RIPA buffer (Sigma). Protein was separated on 10% Bis-Tris gels (Invitrogen), using 15 μg of CD4+ T-cell protein per lane. After electro-blotting transfer to PVDF membrane (Millipore), membranes were blocked for 1 h in 1 x TBST + 5% milk, then probed with anti-THEMIS (ab129174 [EPR7354], abcam) and anti-β-actin (AC-15, Sigma) overnight at 4°C. Blots were incubated with appropriate secondary antibodies (Dako), developed using Pierce ECL reagents (Life technologies), and visualised on a Biorad ChemiDoc Imager. Relative quantification of THEMIS expression to β-actin was performed using Gel analysis functions on Image J.

### Quantification of sjTRECs

The sjTRECs/ml assay was performed according to Lorenzi et al (2008) [35]. Samples were run in triplicate, each reaction contained 500 ng DNA and was supplemented with 25 mM MgCl_2_. Cycling conditions: 94°C 2 min, 40 cycles at 94°C 30 s / 60°C 15 s / 72°C 2 min, 72°C 5 min. CD4+ and CD8+ sjTREC content was determined by calculating sjTRECs/μg DNA; the number of TRECs in 500 ng, a value derived from the sjTREC plasmid standard curve, was multiplied by two.

### *In silico* analysis of transcription factor binding

Data from 1 000 genomes was used to identify SNPs in LD (r^2^ > 0.8; EUR population) with rs13204742. Alggen PROMO was used to predict transcription factor (TF) binding at each SNP plus 25 bp of 5’ and 3’ flanking sequences (assuming binding sites have an average length of 10 bp). The matrix dissimilarity threshold was 5%.

### Statistical analysis

ANOVA or Kruskal-Wallis tests were conducted for all genotypic comparisons, followed by a Turkey’s or Dunn’s multiple comparisons test, respectively. Statistical analyses were performed using GraphPad Prism v6.

## Supporting Information

S1 File Supplementary images A-F.
(DOCX)

S2 File Supplementary Tables A and B.
(XLSX)
