# Supplementary material for: Increased THEMIS First Exon Usage in CD4+ T-Cells Is Associated with a Genotype that Is Protective against Multiple Sclerosis
Source: PLoS One. 2016 Jul 20;11(7):e0158327. doi: 10.1371/journal.pone.0158327 (PMC4954697; doi:10.1371/journal.pone.0158327)
Supplement: S1 File — Supplementary images A-F. (DOCX) [file pone.0158327.s001.docx]

**
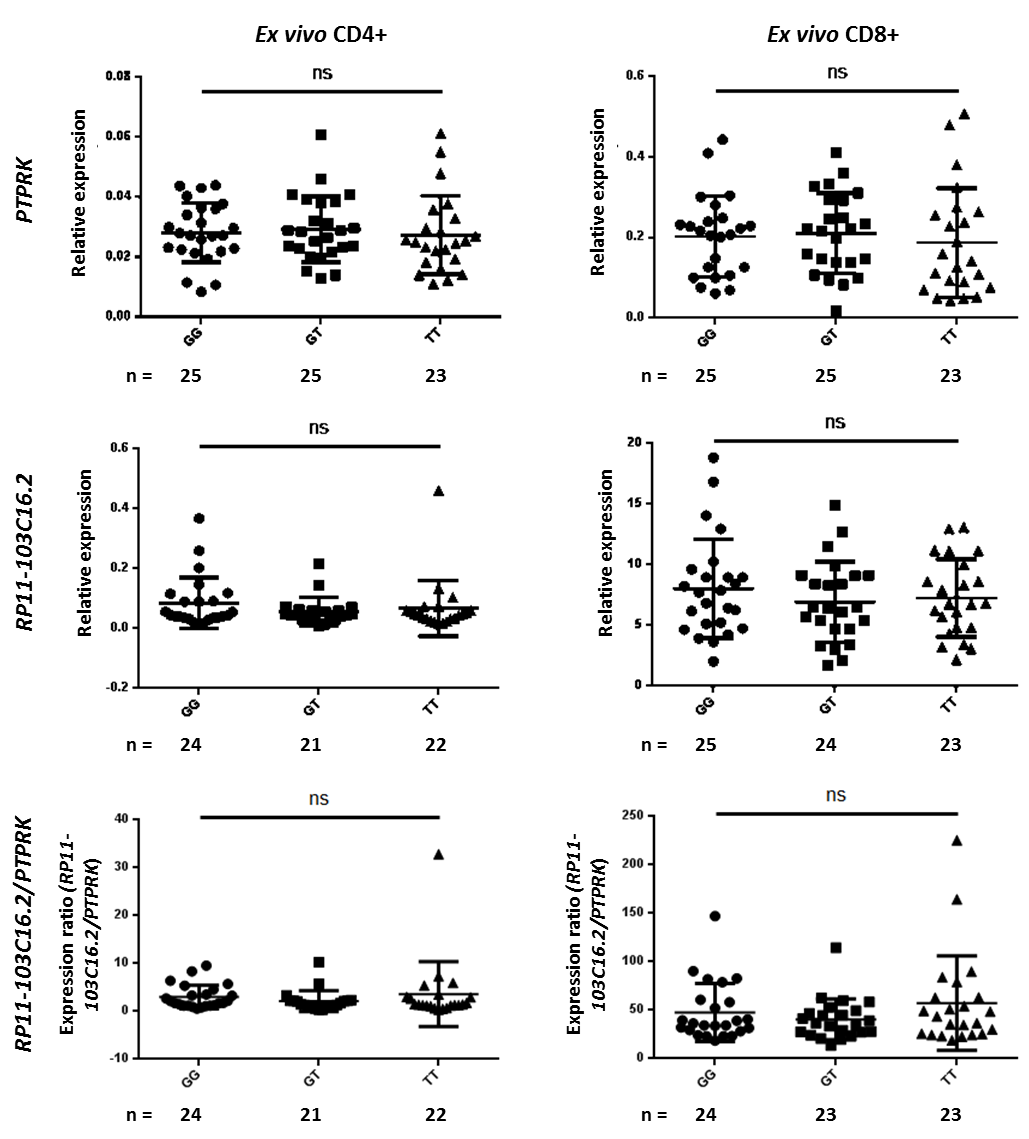
**

**Figure A: There is no association between genotype at SNP rs13204742 and *PTPRK* or *RP11-103C16.2* expression, or the ratio of *PTPRK/RP11-103C16.2* expression, in *ex vivo* CD4+ and CD8+ T-cells. ns = non-significant.**


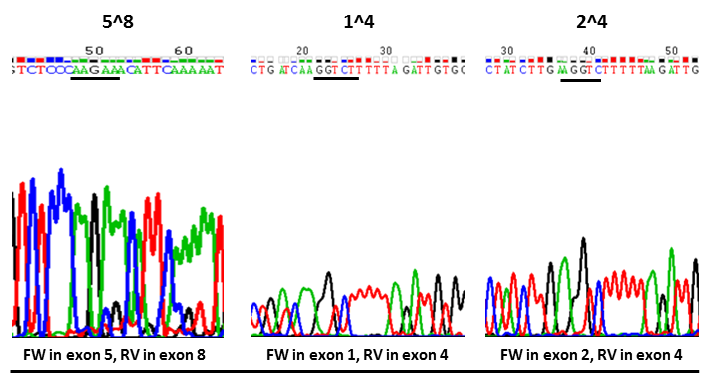


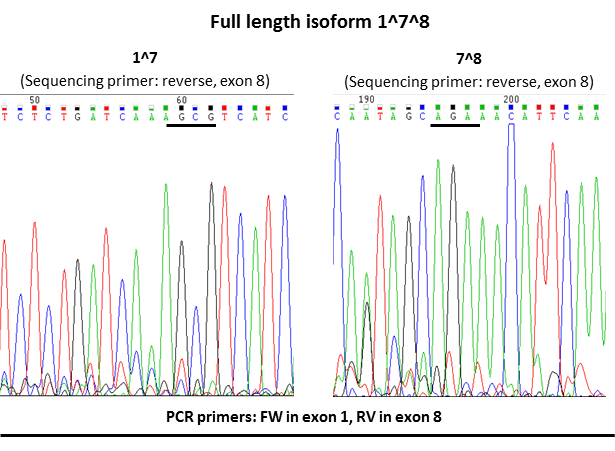


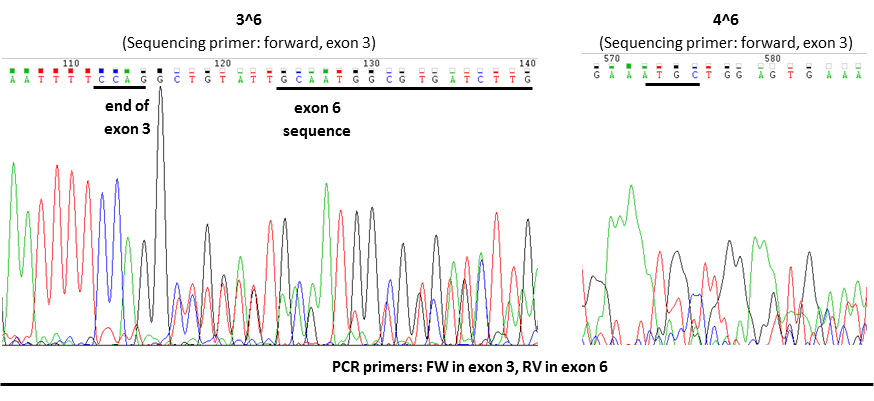


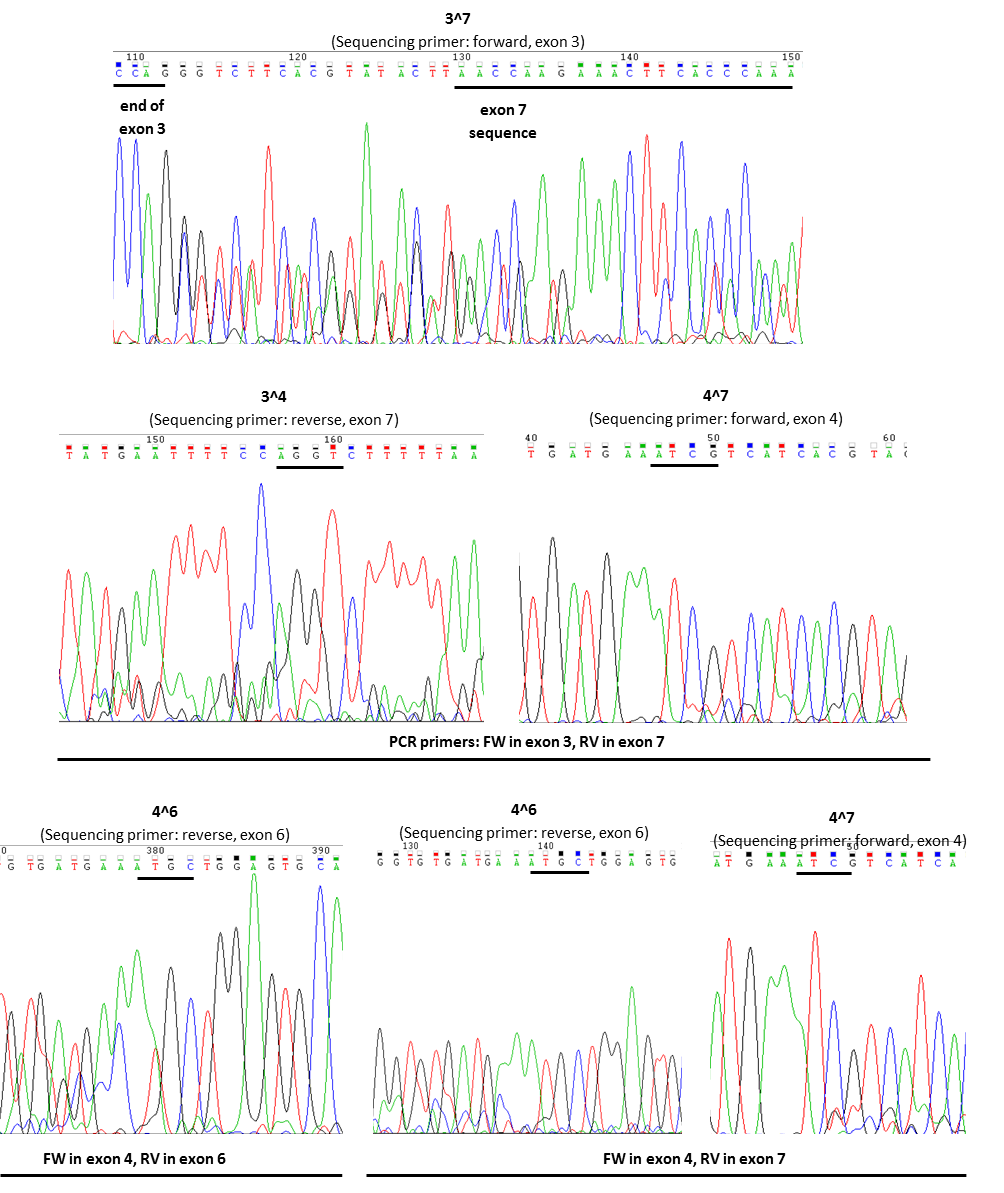


**Figure B: Sequencing chromatograms displaying novel *THEMIS* exon boundaries, identified by end-point PCR. Exon boundaries are indicated by a black line underneath the nucleotide sequence. FW = forward primer; RV = reverse primer; ^ = exon boundary.**

**
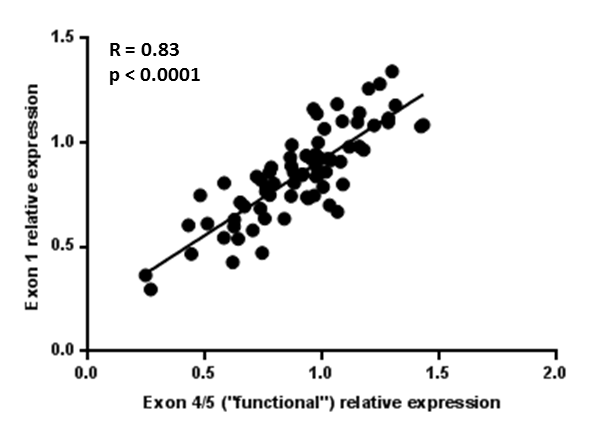
**

**Figure C: There is significant positive correlation between *THEMIS* exon 1 and exon 4/5 expression. The linear regression line and Pearson correlation coefficient are shown.**

**A**


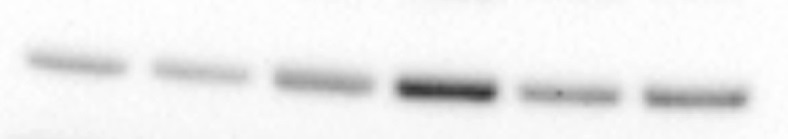


Themis

β-actin


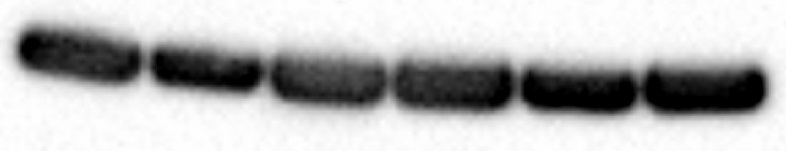


**H**

**H**

**H**

**L**

**L**

**L**

**0.92**

**0.94**

**0.92**

**0.47**

**0.54**

**0.47**

Relative expression
(PCR)

Band density

**1.68**

**2.85**

**0.93**

**1.32**

**0.99**

**1**

Themis mRNA expression status (High/Low)

75 kDa

40 kDa

**A**

**B**

**C**

**Figure D: THEMIS protein expression. (A) *Ex vivo* CD4+ T-cell protein from individuals exhibiting extreme high or low *THEMIS* exon 1 and exon 2 expression (qPCR relative expression values; unpaired t-test p < 0.0001 (B)) were subjected to anti-THEMIS western blotting. The anti-THEMIS antibody was raised to a synthetic peptide corresponding to the THEMIS C-terminus (genomic exon 8), and therefore was assumed to recognise all protein coding THEMIS isoforms. Quantification of relative band densities (Themis vs. β-actin) was performed using Image J; a trend was observed towards greater Themis protein expression with greater *THEMIS* mRNA expression (A). (Unpaired t-test of High vs. Low band density values, p = 0.2764 (C).) (**** p < 0.0001.) Blot images cropped using Image J.**

**C**


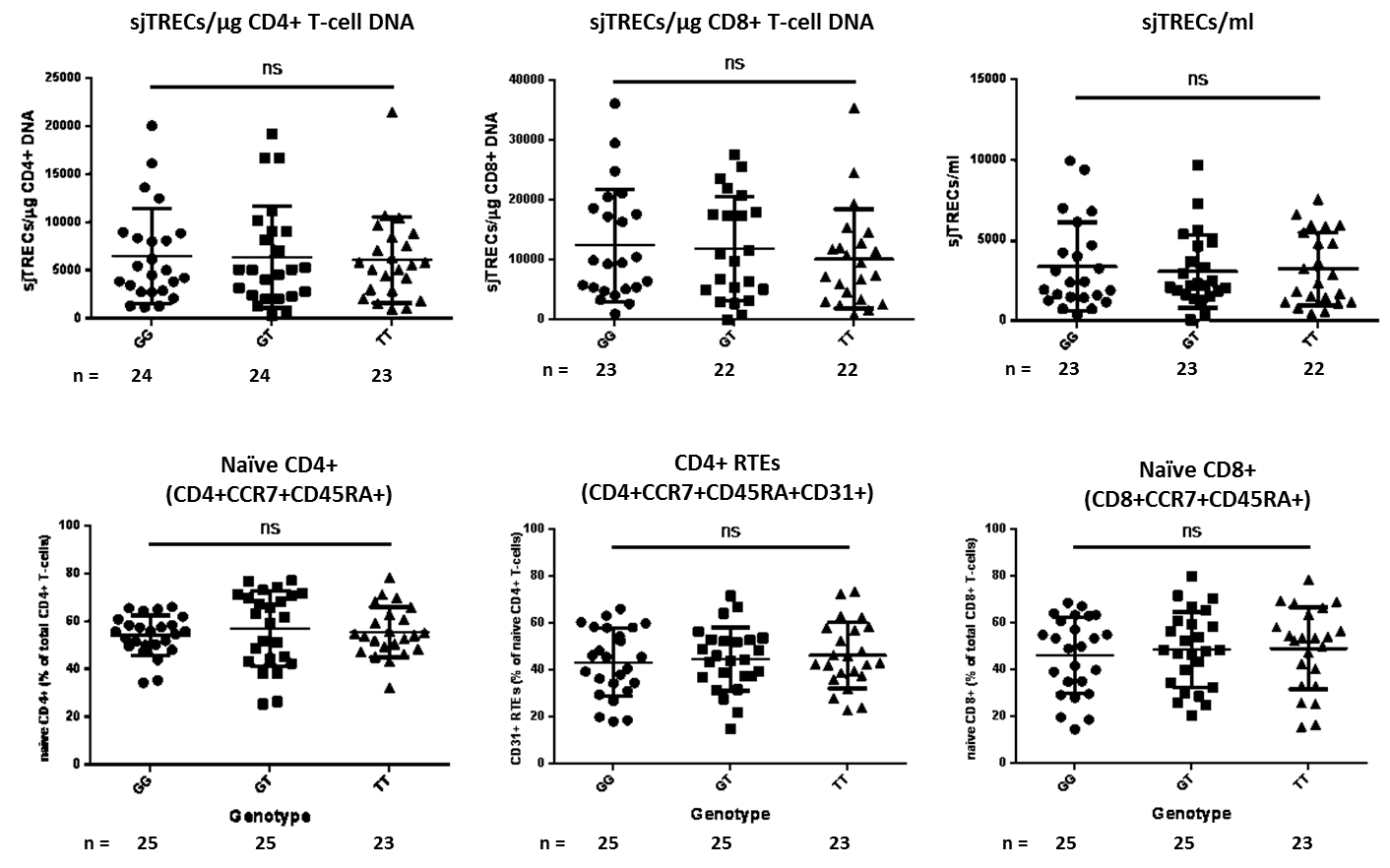


**Figure E: Differences in thymic activity are not associated with genotype at SNP rs13204742; determined by sjTRECs/ml and sjTRECs/µg DNA (top row), and phenotyping of circulating naïve and recent thymic emigrant (RTE) T-cells (bottom row). Error bars display standard deviation from the mean. ns = non-significant.**


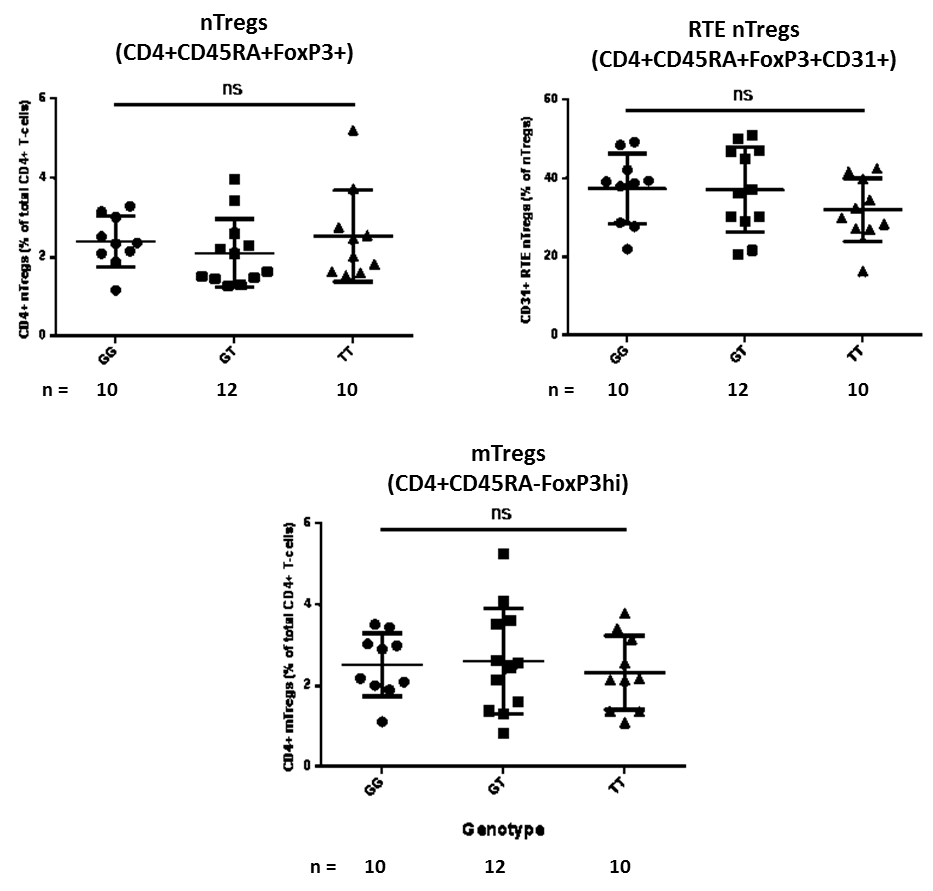


**Figure F: SNP rs13204742 is not associated with differences in the proportions of circulating CD4+ naïve regulatory T-cells (nTregs), recent thymic emigrant nTregs (RTE nTregs), or mature Tregs (mTregs). Error bars display standard deviation from the mean. ns = non-significant.**
